# Gastrointestinal involvement in STEC-associated hemolytic uremic syndrome: 10 years in a pediatric center

**DOI:** 10.1007/s00467-023-06258-5

**Published:** 2024-01-08

**Authors:** Mario Giordano, Onofrio Iacoviello, Luisa Santangelo, Marida Martino, Diletta Torres, Vincenza Carbone, Gaia Scavia, Daniela Loconsole, Maria Chironna, Fernanda Cristofori, Ruggiero Francavilla

**Affiliations:** 1 Pediatric Nephrology and Dialysis Unit, Pediatric Hospital Giovanni XXIII – AOU Consorziale Policlinico, Bari, Italy; 2 Interdisciplinary Department of Medicine, Pediatric Section, University of Bari “Aldo Moro, ” Pediatric Hospital Giovanni XXIII, Bari, Italy; 3 Department of Food Safety, Nutrition and Veterinary Public Health, Istituto Superiore Di Sanità, Rome, Italy; 4 Department of Biomedical Sciences and Human Oncology, Hygiene Section, University of Bari “Aldo Moro”, Bari, Italy

**Keywords:** STEC HUS, Extrarenal involvement, Bowel perforation, Acute pancreatitis

## Abstract

**Background:**

The gastrointestinal (GI) tract represents one of the main targets of typical hemolytic uremic syndrome (HUS) in children. In this observational study, we tried to establish (1) the main features of GI complications during STEC-HUS and (2) the relationship between *Escherichia coli* serotypes and Shiga toxin (Stx) variants with hepatopancreatic involvement.

**Methods:**

A total of 79 STEC-HUS patients were admitted to our pediatric nephrology department between January 2012 and June 2021. Evidence of intestinal, hepatobiliary, and pancreatic involvements was reported for each patient, alongside demographic, clinical, and laboratory features. Frequency of gastrointestinal complications across groups of patients infected by specific *E. coli* serotypes and Stx gene variants was evaluated.

**Results:**

Six patients developed a bowel complication: two developed rectal prolapse, and four developed bowel perforation which resulted in death for three of them and in bowel stenosis in one patient. Acute pancreatitis was diagnosed in 13 patients. An isolated increase in pancreatic enzymes and/or liver transaminases was observed in 41 and 15 patients, respectively. Biliary sludge was detected in three, cholelithiasis in one. Forty-seven patients developed direct hyperbilirubinemia. Neither *E. coli* serotypes nor Shiga toxin variants correlated with hepatic or pancreatic involvement.

**Conclusions:**

During STEC-HUS, GI complications are common, ranging from self-limited elevation of laboratory markers to bowel perforation, a severe complication with a relevant impact on morbidity and mortality. Hepatopancreatic involvement is frequent, but usually short-lasting and self-limiting.

**Graphical abstract:**

**Figure:**
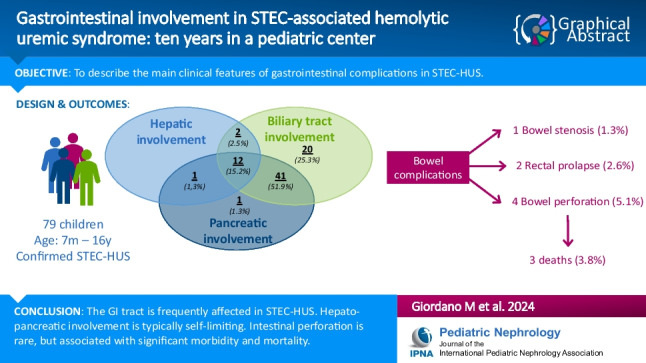
A higher resolution version of the Graphical abstract is available asSupplementary information

**Supplementary Information:**

The online version contains supplementary material available at 10.1007/s00467-023-06258-5.

## Introduction

Hemolytic uremic syndrome (HUS) is a thrombotic microangiopathy (TMA) characterized by thrombocytopenia, non-immune hemolytic anemia and secondary organ damage, and primarily acute kidney injury (AKI). The disease predominantly affects children, with a peak incidence in children under age 5, and is rare in adults [1–3]. We commonly refer to “STEC-HUS” when a GI infection by Shiga-like toxin-producing strains of *Escherichia coli* (STEC) is detected in the setting of established HUS [1, 4].

After STEC-induced bowel infection, children usually develop bloody diarrhea, often associated with abdominal pain, fever, and vomiting. HUS develops in approximately 15% of infected children, mostly younger than 10 and predominantly during summer and autumn. In Italy, O26 is the most commonly identified serotype (38% of cases), while others, such as O157, O145, O111, and O103, are responsible for a minority of cases, different than in other areas of the world [1, 2, 5].

Shiga toxins are encoded by Stx genes, the most common of which are *Stx1a*, S*tx2a*, and S*tx2c*. The toxin (Stx) is produced in the intestine by *E. coli* and translocates across the epithelium through multiple mechanisms [6, 7]. Systemic dissemination follows, leading to platelet aggregation, formation of small thrombi, and intravascular hemolysis due to shear stress to erythrocytes [8, 9]. The kidneys are by far the most severely affected organs during the acute phase of the disease, and HUS is among the leading causes of AKI in children.

Approximately half of affected patients develop additional extrarenal manifestations, and in more than one third of them, the GI tract is involved [10]. Abdominal symptoms such as bloody diarrhea, vomiting, and abdominal pain are expected during STEC-induced gastroenteritis (GE-STEC) [11, 12], while the incidence of other GI complications and their effect on clinical outcome is still poorly documented and is probably underestimated.

This study has two main objectives: (1) Describe the main clinical, instrumental, and laboratory features of intestinal, pancreatic, and liver complications retrospectively reported in a large cohort of STEC-HUS patients admitted over a 10-year period in a single tertiary referral center and (2) establish whether microbiological features (*E. coli* serotype and Stx variants) are associated with hepatic and/or pancreatic involvement.

## Methods

We reviewed the clinical and autoptic records of 79 consecutive patients admitted or transferred to our pediatric nephrology unit between 1 January 2012 and 30 June 2021, with a diagnosis of STEC-induced HUS. The Giovanni XXIII Pediatric Hospital is one of the largest pediatric hospitals in Southern Italy and is a tertiary regional referral center for pediatric nephrology, covering an estimated population of 700,000 children.

We included all patients with a confirmed STEC-HUS diagnosis aged between 0 and 18 years.

STEC-HUS was defined by the presence of thrombocytopenia (platelet count < 150,000/mm^3^ or sharp drop > 50%), hemolytic anemia (schistocytes and/or elevated LDH levels), and evidence of kidney involvement (serum creatinine level > normal limit for age and/or hematuria and/or proteinuria) in a patient with polymerase chain reaction (PCR)-confirmed STEC infection [13].

For each patient, demographic, microbiological, clinical, and instrumental findings were collected in a database. These included age, race, gender, length of hospital stay, STEC serotype, isolated Stx variant, GI co-pathogens detected (if any), symptoms, and both GI and extra-GI complications including acute kidney failure requiring kidney replacement therapy (KRT) and central nervous system (CNS) involvement (defined as new onset of alteration of consciousness, seizures, hypotonia, hypertonia, verbal disorders, vegetative symptoms [14]). All laboratory reference values were related to patient’s age and gender [15]. In our cohort, when KRT was required, continuous venovenous hemofiltration (CVVH) was the modality of choice.

All patients tested positive for STEC in their stools during their hospital stay. PCR was used for microbiological diagnosis. All positive samples were screened for *Stx1*, S*tx2*, and *eae* virulence genes according to Loconsole et al. [3]. Additionally, positive samples were sent to the Italian National Institute of Health (Istituto Superiore di Sanità, ISS, Rome) for confirmation [11].

### Gastrointestinal complications

#### Bowel complications

We defined bowel complication as any de novo event arising during the course of the disease, including rectal prolapse, bowel perforation, and colonic stenosis. Common presenting symptoms of STEC-associated gastroenteritis such as vomiting, diarrhea, or hematochezia were not labeled as “bowel complications.”

#### Pancreas involvement

We included among pancreatic complications both isolated elevations in serum pancreatic enzymes and overt acute pancreatitis. Hyperamylasemia and hyperlipasemia were classified as *mild* or *severe* in presence of values below or above three times the upper limit of normal (ULN), respectively. “Acute pancreatitis” (ACPAN) was diagnosed according to the INSPPIRE (International Study Group of Paediatric Pancreatitis: In search of a cuRE) criteria [16] (modified after the Atlanta criteria) in the presence of at least two of the following: (1) abdominal pain compatible with ACPAN, (2) increase of serum amylase and/or lipase values ≥ 3 times upper limits of normality (ULN), (3) imaging findings consistent with ACPAN [16, 17].

#### Liver and biliary tract involvement

We defined liver involvement as the presence of markers of cytolytic damage: an increase of serum aspartate transaminase (AST) and/or alanine transaminase (ALT) levels ≥ 5 times the ULN was considered *moderate*, while an increase of ≥ 10 times the ULN was considered *severe* [18].

Direct hyperbilirubinemia, gamma-glutamyltransferase (γGT) elevation, or ultrasound evidence of gallstones/biliary sludge was included among signs of biliary tract involvement (Table 3). Abdominal ultrasound was obtained only in some patients, at the discretion of the treating clinician.

#### Statistical analysis

All normally distributed variables are reported as mean ± SD. Median is indicated for not normally distributed variables. Ranges and interquartile ranges (IQR) are also reported when relevant.

Descriptive statistics to test for differences between subgroups of patients included *t* tests for continuous, normally distributed variables and chi-square (*χ*^2^) tests for categorical variables. Statistical analysis was performed using IBM SPSS26.

## Results

### Patients

Over 10 years, 79 patients were treated in our unit for STEC-HUS. All patients were of White/Caucasian origin, and 43 (54.4%) were females. The mean age was 2.8 years [± 2.5 (range, 0.6–16.4)]. The average length of stay (LoS) was 20.4 ± 14.6 days (range, 8–121), irrespective of gender and STEC serotypes (*p* = NS). Clinical and biochemical descriptives for our cohort of patients are provided in Tables 1 and 2.

**Table 1:** Clinical and laboratory findings on admission

Clinical findings on admission		
	N. of patients	(%)
Fever	25	31.6
Diarrhea/vomiting	70	88.6
Hematochezia	39	49.4
Abdominal pain	17	21.5
Jaundice/icterus	15	19.0
Laboratory findings on admission		
	Mean ± SD	Median (IQR 25–75)
Hemoglobin (g/dL)	9.61 ± 1.84	9.49 (8.35–10.75)
Platelets (10^3^/mm^3^)		26 (17.7–48.00)
Creatinine (mg/dL)		2.79 (1.07–4.85)
LDH (IU/L)		3151.00 (1852.00–3993.00)
Albumin (g/L)	28.90 ± 7.19	
WBC (10^3^/µL)	15.76 ± 6.40	
Natremia (mEq/L)	134.04 ± 5.55	

*IQR* interquartile range, *SD* standard deviation

**Table 2:** Summary of demographic and microbiological data and frequency of extra-gastrointestinal complications

Total	79
Age (mean ± SD)	2.8 ± 2.5
Sex (M:F)	36:43
Length of stay (LoS) (mean ± SD)	20.4 ± 14.6
Serotype	
* O26 (%)*	37 (46.8)
* O45 (%)*	2 (2.5)
* O80 (%)*	1 (1.3)
* O103 (%)*	2 (2.5)
* O111 (%)*	14 (17.7)
* O121 (%)*	2 (2.5)
* O145 (%)*	9 (11.4)
* O157 (%)*	3 (3.8)
* O145/O111 (%)*	1 (1.3)
* O126/O111 (%)*	1 (1.3)
* Undetermined (%)*	7 (8.9)
Stx variant	
* Undetermined (%)*	28 (35.4)
* Stx1 (%)*	3 (3.8)
* Stx2 (%)*	31 (39.2)
* Stx1* + *Stx2 (%)*	17 (21.5)
* eae (%)*	55 (69.6)
Dialysis required (%)	42 (53.2)
CNS involvement (%)	13 (16.5)
Exitus (%)	3 (3.8)

*SD* standard deviation

### Microbiological features

STEC infection was confirmed in all 79 patients. Serotypes were identified in 72 patients (91%). The two most common serotypes were O26 in 37 (46.8%) and O111 in 14 patients (17.7%). We found a co-infection by two STEC serotypes in two cases (O26 + O111 and O111 + O145) (Table 2). Stx variants could be isolated in 51 patients. Relative frequencies of Stx variants and *eae* genes are reported in Table 2. No significant correlations could be found between *E. coli* serotypes O26 and O111 and likelihood of hepatic and pancreatic complications (*p* = NS). Similarly, *Stx1*, *Stx2*, and *Stx1Stx2* positive *E. coli* strains were not associated with a greater risk of developing hepatic and pancreatic complications (*p* = NS). Among co-pathogens, *Clostridia* was the most common (9%), followed by *Salmonella enterica*, *Salmonella typhimurium*, *Citrobacter freundii*, and *Adenovirus*, detected in one patient each. Neither co-pathogens nor specific STEC serotypes were found to have an association with specific bowel, pancreatic, or liver complications (*p* = NS).

### Gastrointestinal involvement

Fifty-eight patients (73.4%) presented one or more GI complications, as summarized in Table 3.

**Table 3:** Clinical, laboratory, and instrumental findings of gastrointestinal involvement and relative frequencies

	N. of patients	(%)
Bowel complications		
* Rectal prolapse*	2	(2.5)
* Bowel perforation*	4	(5.1)
* Stenosis*	1	(1.3)
Liver involvement		
Moderate hypertransaminasemia		
* AST*	8	(10.1)
* ALT*	9	(11.4)
Severe hypertransaminasemia		
* AST*	4	(5.1)
* ALT*	4	(5.1)
Biliary tract involvement		
* Direct hyperbilirubinemia*	47	(59.5)
* Biliary sludge/stones*	4	(5.1)
* GGT elevation*	14	(17.7)
Pancreatic involvement		
Acute pancreatitis	13	(16.5)
Mild increase of lipase/amylase ( < 3 times the ULN)		
* Amylase*	14	(17.7)
* Lipase*	6	(7.6)
Severe increase of lipase/amylase (≥ 3 times the ULN)		
* Amylase*	6	(7.6)
* Lipase*	45	(57.0)
Peritoneal effusion	15	(19.5)
Biliary sludge	3	(3.8)
Cholelithiasis	1	(1.3)

*ULN* upper limit of normal

#### Bowel involvement

**Fig. 1:**
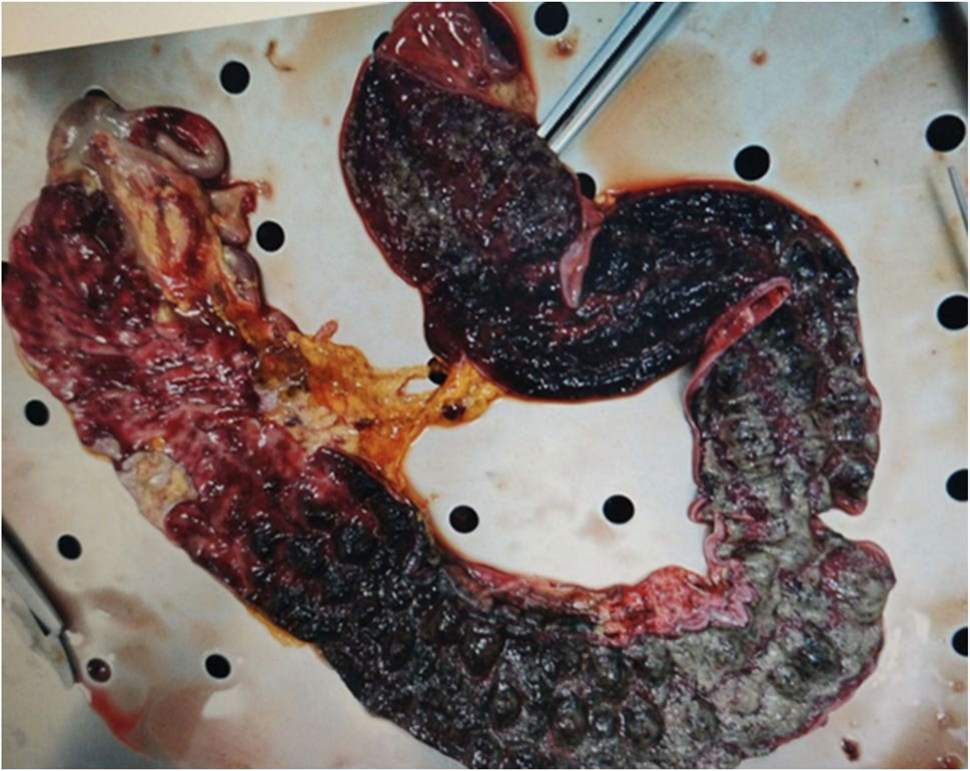
Autoptic findings: hemorrhagic colitis with hemorrhagic and massive necrosis of the intestinal wall

Two patients developed rectal prolapse followed by spontaneous resolution with no relevant sequelae. Four patients developed perforation (patients 1–4). Patients 1 and 3 died 3 days and 12 h after admission, respectively. In both, perforation was confirmed on autopsy, which revealed hemorrhagic colitis with an intraluminal collection of hemorrhagic material and massive necrosis of the intestinal wall (Fig. 1). Patient 3 presented intraluminal pseudomembranous colitis. Patient 2 suddenly developed an intestinal perforation with acute abdominal distension and hemoperitoneum. Patient 4 suffered from bowel perforation 18 days after admission, undergoing emergency surgical resection and temporary sigmoidostomy. Post-operatively, the patient developed segmental stenosis of the colon, requiring surgical resection. The colostomy was reversed by colocolic anastomosis 2 months later. He was the only patient who survived intestinal perforation in our cohort. Fifteen patients (19.5%) presented ultrasound evidence of peritoneal effusion (Table 3).

#### Pancreatic involvement

Thirteen patients (16.5%) met the INSPPIRE criteria for diagnosing ACPAN. In 41 patients (68.4%), we observed an isolated increase in amylase, lipase, or both. This was severe in 32 patients (40.5%). In particular, 4 patients had a severe increase in both, 28 only a severe increase in lipase, and one only increased amylase (Table 3). Serum levels of pancreatic enzymes in affected patients are reported in Table 4. When present, pancreatic enzyme elevation was longer lasting for lipase than it was for amylase. No patient developed endocrine pancreas insufficiency, nor required pancreatic enzymes replacement.

**Table 4:** Duration of elevation of hepatic and pancreatic markers among affected patients

	IU/L	Days		
		*S*	*M*	Return to normal
*AST*	352 (224.0–471.5)	1.0 (1.0–2.0)	1.5 (1.0–3.5)	7.5 (5.3–8.3)
*ALT*	278 (249.0–336.0)	1.0 (1.0–1.8)	1.0 (1.0–2.0)	7.0 (6.0–8.0)
*Amylase*	217 (147.8–317.9)	2.0 (1.3–5.8)		4.5 (3.3–9.8)
*Lipase*	468 (140.0–1273.0)	7.5 (1.8–16)		14.5 (4.0–22.3)

All data are indicated as median (IQR 25–75)

S, number of days with markers in the “Severe” range; M, number of days with markers in the “Moderate” range

#### Hepatic and biliary tract involvement

Fifteen patients had at least a moderate increase in AST and/or ALT. AST elevation was moderate in 8 patients and severe in 4 patients, while ALT elevation was moderate in 9 patients and severe in 4 patients (Table 3). Serum levels of AST and ALT in affected patients, as well as duration of elevation, are reported in Table 4.

Indirect hyperbilirubinemia developed in most patients: 47 patients (59.5%) presented increased conjugated bilirubin, and 15 had jaundice on admission. Fourteen patients (17.7%) showed γGT elevation, one had ultrasound evidence of cholelithiasis, and three had biliary sludge.

We found no differences in terms of age and/or sex between patients presenting with pancreatic (acute pancreatitis or increase of amylase and/or lipase) and/or liver involvement compared to those who did not develop any of those (*p* = NS).

## Discussion

Our study shows a high prevalence of gastrointestinal involvement in children with STEC-HUS ranging within a broad spectrum of clinical manifestations and severities. Hepatobiliary and pancreatic involvements are especially frequent. The clinical relevance of GI complications should not be underestimated. Indeed, in contrast to kidney function, which can be temporarily replaced with KRT (i.e., peritoneal or extra-corporeal dialysis), when other organs are affected, this might worsen the prognosis.

While cardiac [19–21] and neurological [14, 22] involvements in STEC-HUS have been previously described, we could only find a few publications having extraintestinal GI involvement as their primary focus [10]. The pancreas may be affected in multiple ways, potentially leading to acute pancreatitis and pancreatic necrosis, and if the damage is persistent and becomes chronic, can lead to insulin-dependent diabetes [11, 20]. In our cohort, more than half of patients presented evidence of pancreatic involvement, which was severe in 57% of our patients. Lipase elevation appears to be more frequent as well as longer lasting compared to amylase elevation (Table 4). Some degree of amylase and/or lipase elevation in the setting of acute or chronic kidney failure is to be expected, as pancreatic enzymes are partially cleared by the kidneys [23]. This implies that some patients with a mild to moderate increase in pancreatic enzymes possibly do not in fact experience any pancreatic damage, and serum levels of pancreatic enzymes were instead the consequence of reduced renal clearance. In our cohort, ACPAN was self-limited, and no affected patient required specific therapy (i.e., pancreatic enzyme replacement therapy). Diabetes development is a rare complication of STEC-HUS (far less common than acute pancreatitis) and is usually transient [10]. In our cohort of patients, none developed diabetes or sustained hyperglycemia requiring insulin administration. Similarly, despite 13 patients presenting acute pancreatitis, none resulted in exocrine pancreas insufficiency (thus pancreatic enzymes replacement therapy was not indicated).

Hepatic involvement was also common, similarly to what has been previously reported [3, 11, 24], and elevation of liver transaminases was the most frequent manifestation. In most cases, it was self-limited and relatively short lasting, with transaminases returning within the range of normality after approximately 1 week (Table 4).

Unconjugated hyperbilirubinemia, which affected most of our patients, is due to the rapid rate of intravascular hemolysis. In contrast, conjugated (direct) hyperbilirubinemia results from bilirubin buildup and can lead to cholestasis, biliary sludge, and cholelithiasis. In our cohort, three patients were found to have biliary sludge and one had gallstones. However, only 39 patients (49%) underwent abdominal ultrasound, possibly underestimating the prevalence of these among STEC-HUS patients.

The secondary aim of our study was to assess whether some microbiological features, including *E. coli* serotypes and Stx variants, were associated with a greater risk of developing hepatopancreatic involvement. Some Stx variants (i.e., *Stx2a*) have been associated with greater virulence, carrying both a greater risk of causing HUS, as well as resulting more frequently as AKI and KRT [25, 26].

In our cohort, however, neither serotypes (O26 or O111) nor Stx variants were associated with statistically significant differences in the prevalence of hepatic and pancreatic involvement.

The precise mechanism of bowel damage is multifactorial. Two distinct pathological processes have been hypothesized: a vascular mechanism characterized by a microangiopathic thrombosis and a Stx toxin-mediated pro-apoptotic mechanism [27]***.*** These are not mutually exclusive, and both probably contribute to cell injury*.* The involvement of the intestine begins as mucosal ulcerations with some areas of necrosis and progresses to pseudomembrane formation and transmural infarction. If not promptly treated, this can evolve into toxic megacolon, perforation, and hemoperitoneum [20, 27]. Hemorrhagic colitis represents one of the most threatening complications of typical HUS, with a reported mortality as high as 33% according to some authors [28]. In our experience, all three patients who died had developed bowel perforation possibly secondary to hemorrhagic colitis. In all three patients, diagnosis and hospital admission were delayed. “Pain” can be a problematic symptom to interpret, especially in young children or when there is an altered state of consciousness. The threshold for repeat radiological investigations (two projections abdominal X-ray or upright chest radiography to detect subdiaphragmatic air) should be low, and surgical consultation should be pursued early in any patient with severe abdominal pain, acute abdomen, or sudden clinical deterioration. Prompt clinical recognition can increase the chance of survival, as happened with Patient 4.

This study has several strengths: it includes one of the largest cohorts in the literature for any study focusing on gastrointestinal complications in children affected by STEC-HUS. Additionally, it provides a description of the main features of liver and pancreatic involvement, including prevalence, severity of elevation of serum biomarkers, and duration.

We were unfortunately unable to determine how these complications affect prognosis, and this, we believe, represents the greatest limitation of our study. A significantly larger sample of patients would be required in order to establish the contribution that each individual GI complication has on mortality, length of stay, and other relevant prognostic indicators.

## Conclusions

Gastrointestinal complications are common in children affected by STEC-HUS and can involve any organ. Patients with GI involvement should be distinguished from those with common gastroenteritis symptoms that characterize the prodromal phase of STEC infection. Pancreatic and hepatic involvements are common; however, they are often self-limited and resolve in a few days to weeks.

Although rare, intestinal perforation is potentially associated with significant morbidity and mortality. The rapidly evolving nature of the disease, associated with its unpredictability, underlines the need for frequent clinical re-evaluation that could significantly benefit from a multidisciplinary approach (gastroenterologist, nephrologist, radiologist, surgeon, and intensivist). Instrumental investigations (ultrasonography, abdominal X-ray, and CT scan) to identify signs of perforation of intra-abdominal collections must not be procrastinated in patients with sudden clinical deterioration, in order to promptly identify surgical conditions.

More studies are needed to identify the prognostic impact of GI involvement on STEC-HUS patients and to investigate the potential role of laboratory markers such as fecal calprotectin [29] or lactoferrin [30], which could potentially predict intestinal involvement.

### Supplementary Information

Below is the link to the electronic supplementary material.

Graphical abstract (PPTX 45 KB)

## Data Availability

All data regarding this study are included in this published aricle.
