# Supplementary material for: Gastrointestinal involvement in STEC-associated hemolytic uremic syndrome: 10 years in a pediatric center
Source: Pediatr Nephrol. 2024 Jan 8;39(6):1885–91. doi: 10.1007/s00467-023-06258-5 (PMC11026196; doi:10.1007/s00467-023-06258-5)
Supplement: Supplementary file 1 — Graphical abstract (PPTX 45 KB) [file 467_2023_6258_MOESM1_ESM.pptx]

## Slide 1
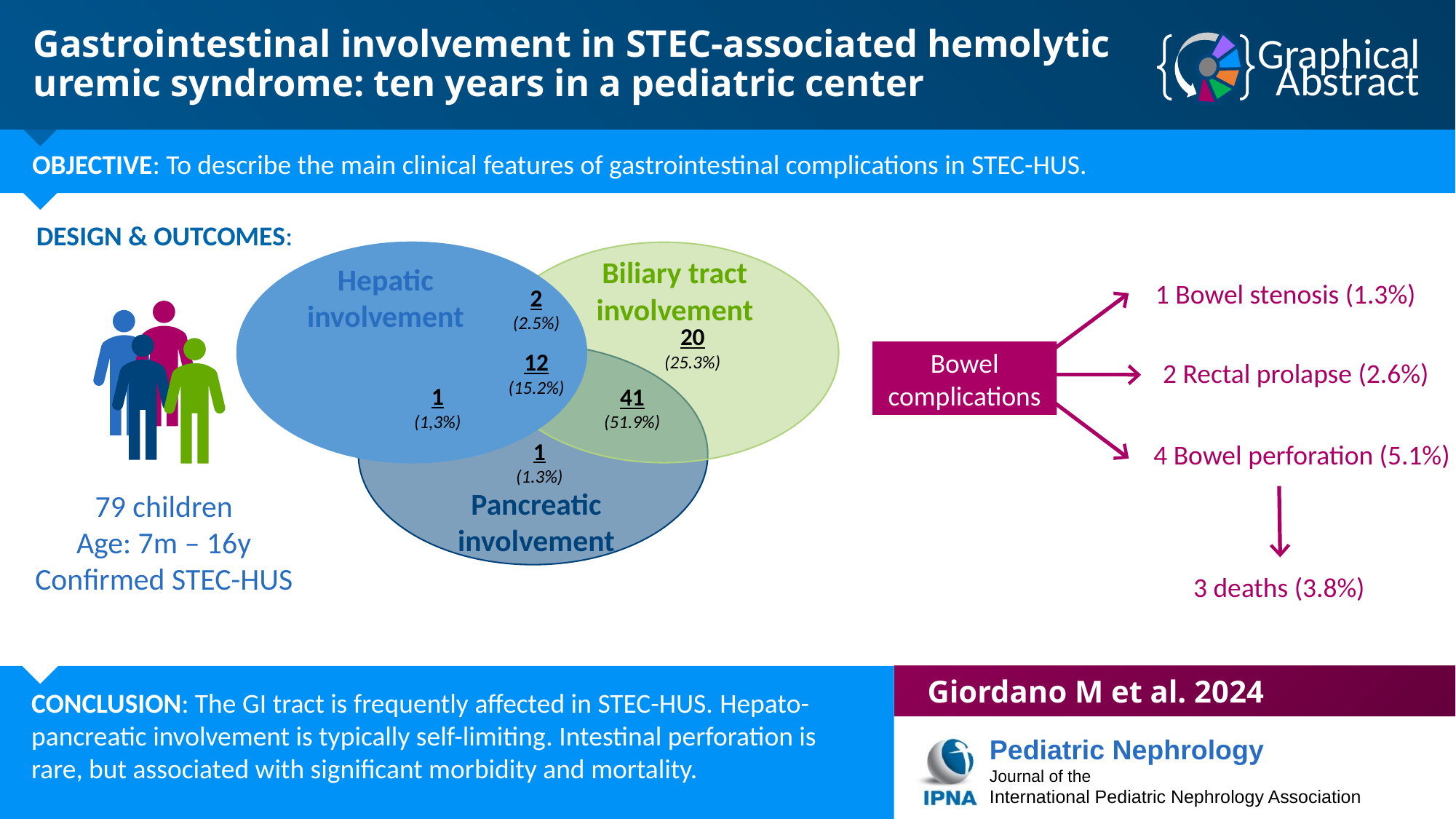

Gastrointestinal involvement in STEC-associated hemolytic uremic syndrome: ten years in a pediatric center
OBJECTIVE: To describe the main clinical features of gastrointestinal complications in STEC-HUS.
DESIGN & OUTCOMES:
Biliary tract involvement
Hepatic involvement
1 Bowel stenosis (1.3%)
2 (2.5%)
20 (25.3%)
12 (15.2%)
Bowel complications
2 Rectal prolapse (2.6%)
1 (1,3%)
41 (51.9%)
1 (1.3%)
4 Bowel perforation (5.1%)
Pancreatic involvement
79 children
Age: 7m – 16y
Confirmed STEC-HUS
3 deaths (3.8%)
Giordano M et al. 2024
CONCLUSION: The GI tract is frequently affected in STEC-HUS. Hepato-pancreatic involvement is typically self-limiting. Intestinal perforation is rare, but associated with significant morbidity and mortality.
